# Detection toward early-stage thermal runaway gases of Li-ion battery by semiconductor sensor

**DOI:** 10.3389/fchem.2025.1586903

**Published:** 2025-04-04

**Authors:** Zhengfu Teng, Cheng Lv

**Affiliations:** Chongqing Water Resources and Electric Engineering College, Chongqing, China

**Keywords:** thermal runaway, semiconductor gas sensor, sensing mechanisms, Li-ion bateries, sensing

## Abstract

While achieving remarkable commercial success, lithium-ion battery (LIBs) carry substantial safety risks associated with potential thermal runaway during widespread applications. When operated under complex working conditions, particularly in high-temperature and high-pressure environments, the internal galvanic reactions within these batteries may escalate uncontrollably. During the early stages of LIBs thermal runaway, substantial amounts of characteristic gases such as H_2_, CO, and CO_2_ are released. Safety assess ent of current thermal runaway status can be achieved through detecting these indicative gas concentrations, thereby enabling efficient and safe utilization of LIBs. This study provides a mini review of current research on semiconductor sensors for detecting early characteristic gases in LIBs thermal runaway through two key dimensions. Firstly, the mechanisms governing the entire thermal runaway process are elucidated, with explicit analysis of gas generation patterns and detectable gas speciation. Subsequently, the review categorically examines research progress on sensors targeting four critical gas categories: carbon oxides, hydrogen, hydrocarbons, and volatile electrolytes. This work establishes a theoretical framework and technical reference for researchers in related fields to advance sensor development, while also providing actionable recommendations to facilitate the fabrication of high-performance sensing devices.

## Introduction

As the first metallic element in the alkali metal group, lithium possesses the smallest ionic radius and lowest density among alkali metals, while exhibiting the highest energy density, endowing it with significant potential in energy storage applications (Wen et al., 2020). Since the commercialization of lithium-ion batteries (LIBs) in 1991 (Reddy et al., 2020), continuous innovations and expansions have been implemented in this battery technology. While achieving remarkable commercial success, LIBs carry substantial safety risks associated with potential thermal runaway during widespread applications. When operated under complex working conditions, particularly in high-temperature and high-pressure environments, the internal galvanic reactions within these batteries may escalate uncontrollably (Song et al., 2024). This process generates excessive Joule heat, ejects copious amounts of toxic and flammable gases, and induces surface deformation through elevated external pressure (Zhang et al., 2021). In severe cases, these cumulative effects can culminate in combustion and explosive events, resulting in catastrophic loss of life and property damage. To prevent and mitigate the loss of life and property damage caused by LIBs thermal runaway, early warning of li-ion battery thermal runaway is of paramount importance. Zhang et al. (2024b) demonstrated that CO, CO_2_, and dimethyl carbonate (DMC) were observed to be emitted from LIBs prior to the onset of thermal runaway under both overcharge and over-discharge conditions. Wang et al. (2024a) discovered that H_2_ is released earlier than CO and CO_2_ in LFP batteries under overcharge conditions. Koch et al. (2018) established that CO_2_, CO, and H_2_ collectively constitute the predominant gaseous components during thermal runaway events in LIBs employed in electric vehicle applications. The implementation of real-time monitoring for characteristic gaseous species coupled with threshold-triggered alert mechanisms has been demonstrated as a reliable methodology for early detection of LIBs thermal runaway (Xu et al., 2024). This approach capitalizes on the continuous surveillance of signature gas emissions, initiating immediate warnings when concentration levels exceed established safety parameters, thereby enabling proactive intervention in energy storage system management (Wang et al., 2024b).

Current methodologies for monitoring lithium-ion battery thermal runaway primarily rely on multi-parameter surveillance through Battery Management Systems (BMS). A BMS integrates heterogeneous sensors (such as: voltage, current, and temperature detectors) to perform integrated analysis of collected signals for real-time operational state assessment (Koseoglou et al., 2020). Notably, during early-stage thermal runaway events, gaseous signatures emerge earlier than voltage, current, or temperature anomalies, while providing more precise indications of incipient failure (Pan et al., 2024; Lai et al., 2025). Consequently, advancements in gas sensor technologies may significantly enhance the detection accuracy of BMS in identifying thermal runaway progression.

When gas molecules interact with the surface of semiconductor materials, charge transfer at the interface induces a contact potential, thereby modulating the electronic structure and altering the electrical properties of the material (Tang et al., 2022; Lu et al., 2024b). Macroscopically, this manifests as either an enhancement or reduction in electrical conductivity upon gas adsorption (Galstyan et al., 2022; Liu et al., 2024). Semiconductor-based detection methods exploit this principle by utilizing materials responsive to the presence of target gases, which are engineered into gas sensors capable of reflecting gas concentrations through variations in electrical signal intensity (Gao and Zhang, 2018; Chen et al., 2024a). Among diverse gas detection techniques, such as Fourier transform infrared spectroscopy (FTIR), catalytic combustion method, and chromatographic method, semiconductor-based approaches are frequently prioritized due to their excellent performance, abundant material availability, and low cost (Wu et al., 2024; Zhang et al., 2024a). This methodology has been extensively applied in early-stage thermal runaway gas detection for LIBs, such as: Wang et al. (2024c) fabricated a ZIF-8-loaded Ag/ZnO nanofiber-based H_2_ gas sensor via a synergistic approach combining electrospinning with a self-sacrificial template strategy. This sensor demonstrated real-time safety warning capabilities, detecting hydrogen leakage 67.79 s prior to LIBs bulge. Hou et al. (2025) successfully synthesized a Ce-doped MoS_2_ H_2_ gas sensor through hydrothermal synthesis method, demonstrating the capability to detect LIBs thermal runaway events within 26 s of their initiation through real-time hydrogen monitoring. Pan et al. (2024) established a novel Bi_2_O_3_ nanosheet-structured DMC gas sensor through hydrothermal synthesis, attaining exceptional sensitivity (50 ppb detection limit) with >15-min preemptive warning capability for LFP battery thermal management. Current researchers are exploring the application of diverse semiconductor materials for detecting early characteristic gases of thermal runaway, while employing multiple materials modification approaches including surface functionalization, material engineering, and composite heterojunction construction to enhance the sensing performance of existing sensors towards thermal runaway gases (Yuan et al., 2022; Lu et al., 2024a). Given the demonstrated potential of semiconductor gas sensors in early warning systems for LIBs thermal runaway events, in-depth investigations into the operational principles and application methodologies of these sensors in this domain are critically warranted.

This work systematically examines the current research landscape of semiconductor sensor-based early gas detection for LIBs thermal runaway through two primary components. Firstly, it introduces the fundamental gas generation mechanisms in LIBs and analyzes detectable gases across progressive thermal runaway stages. Subsequently, the discussion focuses on characteristic thermal runaway gases—including H_2_, CO, and DMC—to evaluate the sensing performance and operational principles of semiconductor sensors targeting these species. The concluding section synthesizes key findings and proposes potential research trajectories. This work provides a theoretical framework and technical references for future investigations while delineating critical directions for advancing this field.

### Gas generation mechanism

The internal thermal runaway process in LIBs, regardless of their specific type, can be categorized into four distinct stages (Yang et al., 2023; Qi et al., 2024a): 1. Initial reaction phase. 2. SEI breakdown phase. 3. Gas ejection phase. 4. Thermal runaway phase. Due to variations in the reactants involved during distinct stages of LIBs thermal runaway, the composition and concentration of evolved gases exhibit marked differences across these phases. For example, during thermal runaway events in LFP batteries, the characteristic gas composition was quantified as follows: H_2_ dominated at 36.34%, followed by CO_2_ at 25.24%, total olefins at 18.87%, total alkanes at 9.94%, CO at 7.39%, with residual gases accounting for 0.73% of the total emissions (Chen et al., 2025). During thermal runaway events in NCM811 batteries, the gaseous emission profile exhibited the following composition: CO constituted the predominant component at 49.26%, followed by H_2_ at 25.06%, CO_2_ at 18.08%, CH_4_ at 5.22%, C_2_H_4_ at 1.62%, with residual gases accounting for 0.76% of the total emissions (Lin et al., 2024).

Initial reaction phase: During the initial reaction phase, the interactions among the anode material, SEI layer, and electrolyte predominantly drive gas evolution. This process is primarily attributed to the thermal decomposition of the SEI layer at elevated temperatures, liberating minor quantities of C_2_H_4_, CO_2_, and O_2_ (Lin et al., 2024), as represented by Equation 1. Concurrently, the temperature elevation induces thermal volatilization of the electrolyte, whose primary constituents—ethylene carbonate (EC), DMC, and diethyl carbonate (DEC) (Qi et al., 2024b), as formalized in Equation 2:

$${\left(CH_{2}OCOLi\right)}_{2}\to LiCO_{3}+C_{2}H_{4}+CO_{2}+O_{2}$$ (1)

$$EC\left(aq\right)+DMC\left(aq\right)+DEC\left(aq\right)+...\to EC\left(g\right)+DMC\left(g\right)+DEC\left(g\right)+...$$ (2)

SEI breakdown phase: With progressive temperature escalation and concomitant decomposition of SEI, the anode becomes exposed to the electrolyte, establishing direct contact with the cathode (Zhang et al., 2022a). This configuration significantly enhances electron transfer kinetics, thereby accelerating the electrocatalytic decomposition of the electrolyte. Concurrently, reactive constituents within the electrolyte interact with lithium dendrites, generating substantial quantities of Hydrocarbons—CH_4_, C_2_H_4_ and C_3_H_6_—as thermodynamically formulated in Equations 3–5 (Zou et al., 2022):

$$Li+C_{3}H_{6}O_{3}\left(DMC\right)\to C_{2}H_{3}O_{3}Li+CH_{4}$$ (3)

$$\text{Li}+C_{3}H_{4}O_{3}\left(EC\right)\to Li_{2}CO_{3}+C_{2}H_{4}$$ (4)

$$Li+C_{3}H_{6}O_{3}\left(DMC\right)\to Li_{2}CO_{3}+C_{2}H_{6}$$ (5)

Gas ejection phase: Upon progression of the reaction to its critical stage, SEI undergoes catastrophic decomposition, resulting in large-scale exposure of the anode to the electrolyte. This triggers vigorous exergonic reactions between the anode, residual SEI fragments, and electrolyte components. These reactions liberate substantial quantities of characteristic gaseous products—predominantly CO_2_, and H_2_ (Chen et al., 2025). Concomitantly, as the thermal runaway progression reaches extreme temperatures (>200°C), the electrolyte decomposition approaches completion through a self-accelerating oxidative decomposition mechanism. This process not only sustains olefin evolution but also generates significant quantities of H_2_, CO_2_ and CO, primarily originating from dehydrogenation of carbonate-based solvents and oxidation of residual SEI constituents, respectively (Galushkin et al., 2024). The corresponding chemical reaction equations are provided in Equations 6–9.

$$LiC_{6}+HF\to LiF+H_{2}+C_{6}$$ (6)

$$Li_{2}CO_{3}+PF_{5}\to LiF+POF_{3}+CO_{2}$$ (7)

$$C_{3}H_{6}O_{3}\left(DMC\right)+O_{2}\to CO_{2}+CO+H_{2}$$ (8)

$$C_{3}H_{4}O_{3}\left(EC\right)+O_{2}\to CO_{2}+CO+H_{2}$$ (9)

**FIGURE 1:**
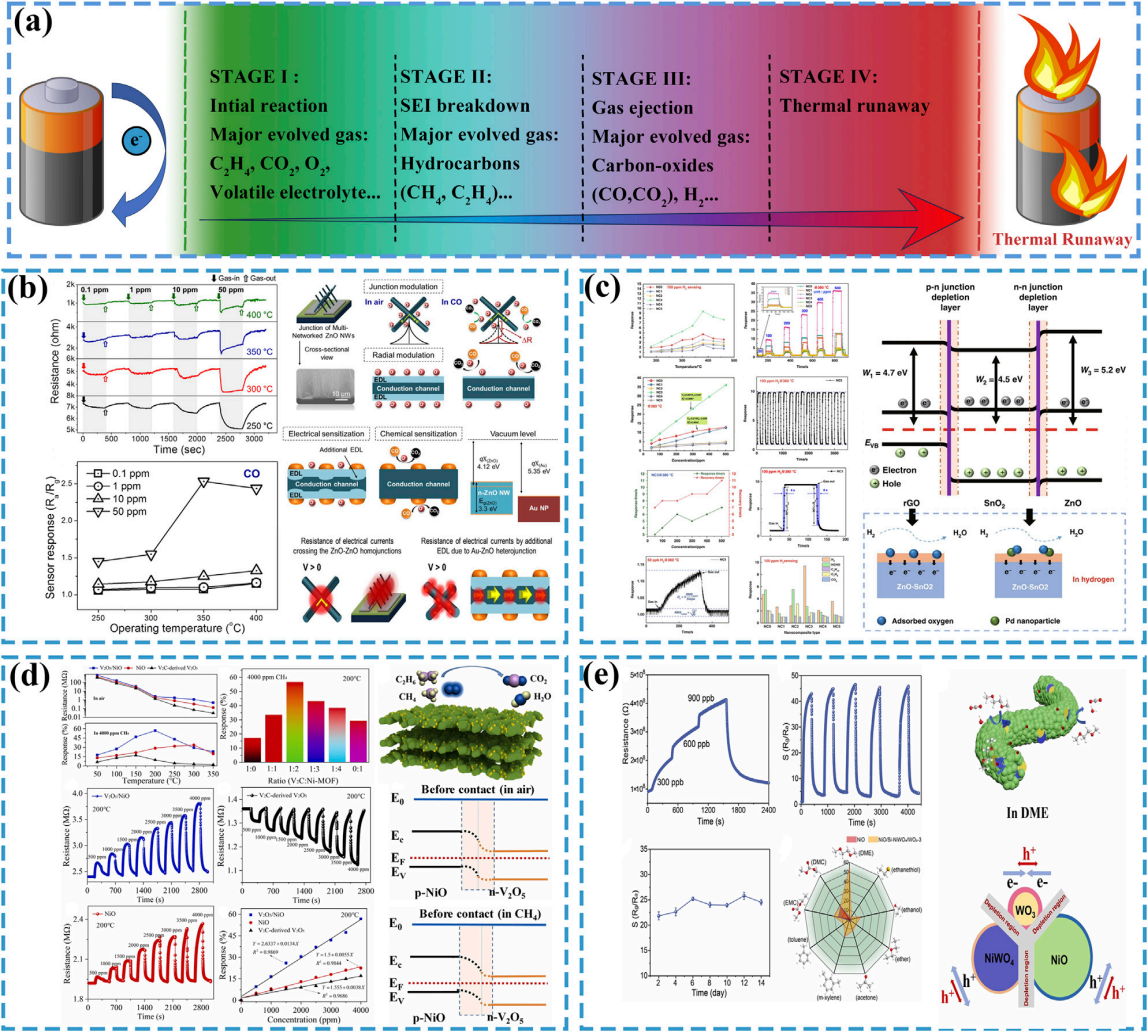
**(a)** The thermal runaway process and evolved gas components in LIBs. **(b)** The CO sensing performance and gas-sensing mechanism of Au-ZnO NWs, reused with permission from (Kim et al., 2019). **(c)** The H_2_ sensing performance and gas-sensing mechanism of Pd-rGO/ZnO-SnO_2_, reused with permission from (Zhang et al., 2022b). **(d)** Gas-sensing performance and gas-sensing mechanism of V_2_O_5_/NiO toward CH_4_, reused with permission from (Wang et al., 2025). **(e)** Gas-sensing performance and gas-sensing mechanism of NiO/Si-NiWO_4_/WO_3_ toward DME, reused with permission from (Gao et al., 2024).

The thermal runaway process in LIBs fundamentally arises from intricate chemical interactions among the cathode, anode, electrolyte, and SEI layer. This reaction cascade releases substantial energy while generating detectable characteristic gases, including CO, CO_2_, H_2_, and DMC, as schematically illustrated in Figure 1a. Implementing detection systems capable of identifying such gas anomalies prior to thermal runaway enables preventive countermeasures (Chen et al., 2024b). The subsequent chapter will systematically review current research progress in semiconductor sensor technologies for early-stage gas detection during battery failure precursors.

### Gas-specific detection techniques

#### Carbon oxides

As outlined in Section 2, the carbon-oxygen emissions generated during the early stages of thermal runaway in LIBs predominantly consist of CO and CO_2_, which constitute critical components of the gas-phase products associated with such failure events (Yang et al., 2023).

To optimize the CO-sensing capabilities of gas sensors, significant research endeavors have focused on material modification strategies to accomplish this objective. Kim et al. (2019) was synthesized ZnO nanowires (ZnO NWs) via the vapor-liquid-solid growth mechanism. The contact between ZnO and Au induced electron transfer from the conduction band of ZnO to Au, resulting in the formation of a thin electron depletion layer (EDL) at their interface. This interfacial engineering significantly enhanced the CO-sensing performance. As shown in Figure 1b, the optimized sensor demonstrated a response value of 1.015–100 ppb CO under low operational conditions (7 V applied voltage, 300°C working temperature). Baier et al. (2023) fabricated CeO_2_ and Cu-CeO_2_ materials with high specific surface areas and uniform porosity by employing mesoporous silica as a structural template, where the nanocasting technique guided the crystallographic growth orientation. Comparative experimental investigations revealed that the incorporation of Au species improved the CO gas-sensing performance of CeO_2_-based materials, resulting in enhanced stability of baseline resistance and response profiles during CO exposure, while reducing the optimal operating temperature for CO detection from 600°C to 450°C. Naganaboina and Singh (2021) introduced graphene nanoplates (GNPs) into CeO_2_ through a hydrothermal method, constructing heterojunctions dominated by Ohmic contacts between GNPs and CeO_2_ to achieve surface modification of CeO_2_. This incorporation strategy enhanced the charge transport capacity of CeO_2_ without altering its intrinsic n-type semiconductor characteristics, leading to the generation of additional oxygen vacancies on the surface. The GNPs-CeO_2_ composite exhibited sensitive responses to 2 ppm CO under room-temperature conditions, thereby validating the improvement in gas-sensing performance.

The gas-sensing mechanism of CO is fundamentally associated with its inherent reducing properties, which drive the spontaneous electron release from CO molecules and their subsequent oxidation into a thermodynamically stabilized CO_2_ configuration (Naganaboina and Singh, 2021; Piliai et al., 2023). Theoretical models indicate that upon atmospheric exposure, gas sensors initially adsorb atmospheric oxygen molecules and facilitate their conversion into chemisorbed oxygen species (such as: $O_{2}^{-}$ ) through electron donation, thereby depleting the surface charge density. When subsequently exposed to CO, the intrinsic reducibility of CO drives it to undergo a surface redox reaction with the chemisorbed oxygen species (Qin et al., 2025). This interaction generates CO_2_ while releasing electrons back into the sensing material, consequently modulating the electrical properties of the sensor (Zhang et al., 2024c). The reversible nature of the latter reaction enables regeneration of the active sites, establishing a cyclic detection capability for repeated CO response (Wang et al., 2022).

Current research findings systematically reveal that heterojunction architectures exhibit superior CO_2_ sensing performance metrics at lower gas concentration compared to their non-heterojunction counterparts under identical testing protocols (Bolli et al., 2025). Bolli et al. (2025) engineered a p-n SnO-SnO_2_ heterojunction composite via radio-frequency magnetron sputtering deposition of SnO_2_ thin films onto SnO substrates. This architecture demonstrated a 1.07 response toward 1,000 ppm CO_2_ with a sub-1-s response time. Singh et al. (2023) fabricated a p-p CeO_2_/CdS heterostructure via a two-stage hydrothermal synthesis, where CdS nanoparticles were epitaxially grown on CeO_2_ microspheres. Benefiting from the triboelectric nanogenerator characteristics of the composite, the hybrid material exhibited reduced operational voltage requirements and superior sensing performance. The optimized heterojunction demonstrated a gas response factor of 3.96 toward 1,000 ppm CO_2_, with theoretical detection limits reaching 250 ppm.

Notably, given the atmospheric CO_2_ concentration of approximately 400 ppm, current sensor research predominantly focuses on ppm-level detection thresholds (Suzuki et al., 2020). The chemical stability of CO_2_ dictates that its sensing mechanism in semiconductor gas sensors diverges from conventional redox reactions (Haldar et al., 2024; Huang et al., 2025). The detection process initiates with oxygen molecule adsorption on the sensor surface, where oxygen acquires electrons to form chemisorbed oxygen species ( $O^{-}$ or $O_{2}^{-}$ ). Subsequently, CO_2_ interacts with these activated oxygen intermediates, extracting additional electrons to generate charged $CO_{2}^{-}$ adducts and $CO_{3}^{2-}$ . This reaction pathway exhibits inherent reversibility, enabling material regeneration for cyclic sensing operations. The absence of redox transformations during CO_2_ adsorption induces metastable surface binding configurations, which fundamentally limits electron transfer efficiency and consequently reduces detectable signal amplitudes in low-concentration regimes (Zhou et al., 2019). Heterojunction architectures can be engineered through the strategic formation of Ohmic or Schottky contacts, establishing carrier depletion/accumulation layers that fundamentally modulate majority carrier density profiles (Haldar et al., 2024). This interfacial charge redistribution simultaneously enhances charge transfer kinetics and oxygen vacancy formation thermodynamics, thereby amplifying CO_2_ gas-sensing performance via synergistic surface reactivity optimization (Bolli et al., 2025).

#### Hydrogen

Within the thermal runaway mechanism of LIBs, hydrogen evolution predominantly occurs during Stage III, where elevated temperatures trigger intense exothermic chain reactions that persist for 20–100 s (Yang et al., 2023). This operational imperative demand hydrogen sensors with dual functionality: ultrahigh sensitivity for trace-level hydrogen detection and fast response-recovery cycles (Hou et al., 2025). Zhang et al. (2022b) synthesized a Pd-doped ZnO-SnO_2_ material modified with reduced graphene oxide (Pd-rGO/ZnO-SnO_2_) via a hydrothermal method. As shown in Figure 1c, the material exhibited a high sensitivity response to 100 ppm hydrogen gas, with a response time of 4 s and a desorption time of 8 s. The calculated lower detection limit of the modified material for hydrogen reached as low as 50 ppb, demonstrating exceptional hydrogen sensing capabilities. Patrnciak et al. (2025) fabricated a nanoporous Pt/TiO_2_/Pt sensing structure material via electromagnetic sputtering. This material demonstrated a sensitive response to 3 ppm hydrogen at room temperature. Moreover, the sensor retained its capability to detect 10 ppm hydrogen under high-humidity conditions (>60% RH) at ambient temperature.

Hydrogen, as a typical reducing gas, shares a similar sensing mechanism to CO. Hydrogen interacts with adsorbed oxygen on the surface of gas-sensitive materials, reacting to form gas H_2_O molecules and releasing electrons. This process alters the charge transport properties of semiconductor materials, enabling hydrogen detection (Jing et al., 2025). It is well-established that CO exhibits stronger reducibility than H_2_, yet the superior gas-sensing response of materials toward H_2_ stems from their intrinsic capacity to undergo redox reactions with hydrogen, which generally exhibit higher reaction intensity compared to those with other gases (Zhang et al., 2022b). Montoro et al. (2024) developed a MOF-derived ZnO nanomaterial sensor capable of generating gas-sensing signals for H_2_, CO, and H_2_S simultaneously. Experimental results revealed that the MOF-ZnO exhibited higher sensitivity toward H_2_ compared to the other two gases. This enhanced response arises from the preferential reduction of ZnO to metallic Zn with higher charge density upon interaction with H_2_ at operating temperatures, whereas no analogous reactions occurred when exposed to CO or H_2_S.

#### Hydrocarbons

Hydrocarbons generated during thermal runaway of LIBs are predominantly composed of alkanes and alkenes (such as CH_4_ and C_2_H_4_), with their relative proportions varying depending on battery types (Qi et al., 2024a). Accounting for approximately one-quarter of gaseous components in thermal runaway emissions, hydrocarbons are even produced in trace amounts during the initial stage of battery failure (Zou et al., 2022). Detecting these hydrocarbons enables earlier assessment of battery status, thereby facilitating proactive thermal runaway prevention. Nie et al. (2024) synthesized In_2_O_3_-xCuO nano heterojunction materials via a hydrothermal method by compositing In_2_O_3_ with CuO. The material exhibited a gas-sensing response of 1.043 toward 400 ppm methane at an operating temperature of 350°C with a lower detection limit of 50 ppm. Experimental results demonstrated that the material’s methane-sensing capability outperformed its responses to CO and H_2_. Wang et al. (2025) fabricated a V_2_O_5_/NiO nano-hybrid methane sensor by integrating V_2_CT_x_ MXene with Ni-based metal-organic frameworks (MOFs) through an annealing process. As shown in Figure 1d, the sensor did exhibit a gas-sensing response of 1.57 toward 4,000 ppm CH_4_ at 200°C, while demonstrating a lower detection limit of 50 ppm. Furthermore, the research team developed a random forest algorithm model, revealing that the sensor could accurately and efficiently detect CH_4_ within thermal runaway gases of LIBS. Yang et al. (2024) fabricated a self-heating ethylene sensor by depositing SnO_2_ on laser-induced graphene (LIG) via a spin-coating deposition method. The sensor demonstrated an ultralow detection limit of 1.654 ppb for ethylene, a high sensitivity of 0.2249 ppm^–1^, and excellent long-term stability, enabling real-time monitoring of ethylene concentration in confined environments over extended periods.

Alkenes possess reducibility owing to the unsaturated bonds between their internal carbon atoms. Upon contact with adsorbed oxygen species, they simultaneously generate carbon dioxide and water molecules. However, the unstable bonds formed subsequently revert back to the original olefin molecules, thereby achieving the sensing response to alkenes (Yang et al., 2024). Alkanes, particularly methane, form relatively stable chemical structures that are less prone to chemical reactions. The underlying gas-sensing mechanisms remain unclear in the current academic community. One hypothesis suggests that its reaction mechanism resembles that of olefins due to the weak reducibility inherent in methane as a hydrocarbon, while another hypothesis posits that the mechanism is associated with mutual interactions between alkanes and sensor materials (Hamad et al., 2024; Wang et al., 2025).

#### Volatile electrolyte

LIBs electrolytes, including dimethyl carbonate (DMC), ethylene carbonate (EC), and diethyl carbonate (DEC), serve as crucial battery components. These electrolytes exhibit strong reducing characteristics to enable real-time charge transfer within batteries, which also renders them prone to react with sensor materials (Fan et al., 2022). Furthermore, their relatively low melting points allow easy vaporization and escape from batteries once thermal runaway occurs, thus providing detectable conditions for sensors. However, since vaporization primarily occurs during the initial stage of thermal runaway with limited gas emission, sensors are required to respond to ultra-low concentrations (ppb-level) of electrolytic vapors (Zhang et al., 2024b). Wan et al. (2023) fabricated a novel sensing material, Co/Pd-SnO_2_, through an annealing process that co-doped Co and Pd into SnO_2_. This material exhibited exceptional sensing performance toward gaseous DMC, demonstrating a response value of 1.5 toward 500 ppb DMC at an operating temperature of 150°C. In simulated electrolyte leakage scenarios, the sensor achieved a prompt response within 50 s. Gao et al. (2024) fabricated heterostructured NiO/Si-NiWO_4_/WO_3_ nanofiber materials through a two-step method involving electrospinning and thermal oxidation. In practical testing, this sensor demonstrated a high gas-sensing response to dimethoxyethane (DME), achieving a response value of 1.87 toward 300 ppb gaseous DME at an operating temperature of 300°C, as shown in Figure 1e. Sun et al. (2025) fabricated Ag-doped SnS_2_ with nanoflower-like morphology via a hydrothermal method, followed by a calcination process to synthesize Ag@Ag_2_O-SnO_2_. At an operating temperature of 250°C, the sensor demonstrated a response value of 106 toward 100 ppm DMC, accompanied by response/recovery times of 28/55 s and a lower detection limit of 11.76 ppb. As a strongly reducing gas, the electrolyte components can react with adsorbed oxygen on the surface of semiconductor materials, generating CO_2_ and H_2_O. The reversibility of this reaction enables the sensing material to be reusable (Wan et al., 2023; Gao et al., 2024). For systematic comparative analysis, the gas-sensing performance of all mentioned materials is comprehensively summarized in Table 1.

**TABLE 1:** The sensing properties of semiconductor sensor toward thermal runaway characteristic gases.

Gas type	Sensor	Concentrate	Sensitivity (R_g_/R_0_)	Operating temperature	Source
CO	Au-ZnO NWs	100 ppb	1.015	300°C	Kim et al. (2019)
	Cu-CeO_2_	60 ppm	1.004	450°C	Baier et al. (2023)
	GNPs-CeO_2_	2 ppm	1.35	RT	Naganaboina and Singh (2021)
CO_2_	SnO-SnO_2_	1000 ppm	1.07	RT	Bolli et al. (2025)
	CeO_2_/CdS	1000 ppm	3.96	RT	Singh et al. (2023)
H_2_	Pd-rGO/ZnO-SnO_2_	100 ppm	4.73	380°C	Zhang et al. (2022b)
	Pt/TiO_2_/Pt	10,000 ppm	3.1 × 10^9^	RT	Patrnciak et al. (2025)
CH_4_	In_2_O_3_-xCuO	400 ppm	1.043	350°C	Nie et al. (2024)
	V_2_CT_x_/NiO	4000 ppm	1.57	200°C	Wang et al. (2025)
C_2_H_4_	SnO_2_/LIG	10 ppm	1.002	58°C	Yang et al. (2024)
DMC	Co/Pd-SnO_2_	500 ppb	1.5	150°C	Wan et al. (2023)
	Ag@Ag_2_O-SnO_2_	100 ppm	106	250°C	Sun et al. (2025)
DME	NiO/Si-NiWO_4_/WO_3_	300 ppb	1.87	300°C	Gao et al. (2024)

## Summary and outlook

With the continuous expansion of the LIBs application market, real-time monitoring of LIBs holds significant practical importance for ensuring their safe operation and preventing/early-warning thermal runaway incidents. During the early stages of LIBs thermal runaway, substantial amounts of characteristic gases such as H_2_, CO, and CO_2_ are released. Safety assessment of current thermal runaway status can be achieved through detecting these indicative gas concentrations, thereby enabling efficient and safe utilization of LIBs. This work systematically reviews the current research status on semiconductor sensor-based gas detection during early-stage thermal runaway of LIBs, focusing on two key aspects: 1) This study systematically investigates the stage-specific characteristics of LIBs thermal runaway and summarizes the corresponding gas emission profiles. 2) This study categorically analyzes semiconductor sensors for detecting carbon oxides, Hydrogen, hydrocarbons, and volatile electrolytes through the lens of gas speciation, with particular emphasis on their gas-sensing performance and underlying mechanisms. This work establishes a theoretical framework and technical references for researchers in related fields to advance sensor development, while also providing actionable recommendations to propel progress in this domain.
